# Onion Peel Extract Prevents Intestinal Inflammation via AMK-Activated Protein Kinase Activation in Caco-2/HT-29 Cells

**DOI:** 10.3390/nu16213609

**Published:** 2024-10-24

**Authors:** Olugbenga Balogun, Cindi R. Brownmiller, Sun-Ok Lee, Hye Won Kang

**Affiliations:** 1 Applied Science and Technology, North Carolina Agricultural and Technical State University, Greensboro, NC 27411, USA; otbalogun@aggies.ncat.edu; 2 Department of Food Science, University of Arkansas, Fayetteville, AR 72704, USA; cbrownm@uark.edu (C.R.B.); sunok@uark.edu (S.-O.L.); 3 Food and Nutritional Sciences, Department of Family and Consumer Sciences, North Carolina Agricultural and Technical State University, Greensboro, NC 27411, USA

**Keywords:** flavonols, quercetin, intestine, obesity

## Abstract

Background: Obesogenic diets cause intestinal inflammation and dysfunction. Polyphenols have shown a positive impact on reducing inflammation in in vitro studies. However, their bioactivity may not be the same in the in vivo system due to structural alteration by the gastrointestinal digestive process. The purpose of this study was to investigate the anti-inflammatory effect of onion peel and its major bioactive compound, quercetin, in the intestine and further examine the impact of intestinal digestion on this effect. Methods: Onion peel extract (OPE) and quercetin (Q) were digested using gastrointestinal digestive enzymes in vitro and then treated into lipopolysaccharide (LPS)-stimulated Caco-2/HT-29 cells. Genes and proteins related to tight junction, inflammation, and epithelial integrity were measured. Results: OPE and digested OPE (DOPE) had a higher protective effect on LPS-induced tight junction and inflammatory genes and paracellular permeability than Q and digested Q (DQ). DOPE was more effective than OPE, while digestion did not change the activity of Q. The anti-inflammatory effect of OPE and Q with or without digestion was achieved by inhibiting nuclear factor kappa B through AMP-activated protein kinase-activated silent mating-type information regulation 2 homolog 1. Conclusions: It was the first to find that a crude extract, after undergoing gastrointestinal digestion, demonstrated a notably superior anti-inflammatory effect in the cell study, suggesting the consumption of onion peels could potentially yield similar benefits in the human intestine. This discovery underscores the potential of onion peel polyphenols in combating intestinal inflammation, making them a compelling area of research for future therapeutic applications using food byproducts.

## 1. Introduction

A high-fat diet causes an imbalance in gut bacteria, leading to the overgrowth of harmful bacteria [1,2]. These bacteria release lipopolysaccharide (LPS) and reduce the amount of short-chain fatty acids (SCFAs), the bacterial metabolites [1,2]. The LPS increases the production of proinflammatory markers such as tumor necrosis factor-alpha (TNF-α), interleukin-6 (IL-6), and interleukin-1 beta (IL-1β) by activating the nuclear factor kappa-light-chain-enhancer of activated B cells (NFκB), a key mediator of inflammatory regulation [3]. This disrupts tight junctions (TJs), composed of proteins like zonula occludens (ZO-1, ZO-2, ZO-3), occludin (OCLN), claudins (CLDN), and junctional adhesion molecules (JAM) that physically connect adjacent epithelial cells, regulate paracellular water and ion transport, and block luminal toxins and bacteria [4]. The disruption of TJs increases intestinal permeability, which leaks LPS into the bloodstream [4]. This contributes to the development of chronic low-grade inflammation-related diseases, such as obesity [5]. Unhealthy lifestyle habits such as low vegetable intake, high processed meat consumption, and a sedentary lifestyle can also increase the presence of intestinal proinflammatory macrophage subpopulation P2 in the gastrointestinal tract including the stomach, duodenum, and colon [6]. This occurs by recruiting intermediate monocytes into the gut wall, which are not further differentiated into anti-inflammatory macrophages, leading to gut inflammation in obese individuals compared to non-obese individuals [6]. The SCFAs released by gut microbiota play key roles in controlling fat deposition in the host’s body by regulating calorie absorption in the intestine and lipoprotein lipase activity associated with triacylglycerol storage in adipose tissue, modulating NFκB activity, maintaining the integrity of the gut barrier, and reducing LPS-induced accumulation of macrophages in the intestine [1,2]. Therefore, maintaining intestinal integrity and preventing gut inflammation is crucial in mitigating obesity-induced inflammation.

Dietary polyphenols can be protective of the intestinal epithelium [7,8,9,10,11,12]. 6-gingerol and resveratrol, a major phytochemical of ginger and red wine, respectively, decreased intestinal permeability by activating AMP-activated protein kinase (AMPK) in LPS-stimulated Caco-2 cells [7,8]. Similarly, chlorogenic acid, a phenolic acid found in coffee, reversed LPS/palmitic acid-induced intestinal barrier disruption and suppressed inflammatory responses by inhibiting Rho-associated coiled-coil containing protein kinase (ROCK)—myosin light chain kinase (MLCK) signaling pathways and relieving endoplasmic reticulum stress [9]. Berberine, found in barberry, reduced TNF-α induced claudin-1 disassembly and upregulated claudin-2 expression in HT-29/B6 human colon cells by inhibiting NFκB p65 [11]. Naringenin, a major polyphenol in citrus fruits, enhanced TJ formation and barrier integrity in Caco-2 cells [12]. Kaempferol, a natural flavonoid, showed a protective effect on TJ barrier integrity via ZO-2 and CLDN-4 expression and redistribution of claudin-1 and -3 and occludin [10].

Onion peel, a byproduct of onion, contains 20 times higher concentrations of quercetin and higher levels of total phenolic compound, flavonoids, and flavanols than onion bulb, which supports onion peel’s potential health effects [13,14,15,16,17]. Previously, quercetin alleviated obese conditions in high-fat diet-induced obese mice, especially by decreasing inflammation in brown adipose tissue that burns fat by non-shivering thermogenesis, and reverting its function [18,19]. Moreover, quercetin supplementation also reduced the ratio of *Firmicutes* to *Bacteroidetes* and increased the production of SCFAs in the mice [19]. Kim et al. showed that onion peel ethanol extract reduced *TNFA* gene expression in LPS-induced HT-29 cells [20]. Therefore, we speculated that onion peel and quercetin may positively impact the intestine’s health. When polyphenols are ingested, they undergo a gastrointestinal digestive process, which leads to qualitative and quantitative changes in the phenolic composition, decreasing their bioavailability and health effects [21]. Hence, the bioactivity of onion peel found in the cell system may not mirror its impact on the human body. There are no studies to examine the bioactivity of gastrointestinal digested onion peel in vitro to mimic in vivo biological effects. Therefore, this study aims to investigate the anti-inflammatory effects of in vitro gastrointestinal digested onion peel extract and its single compound in Caco-2/HT-29 cells.

## 2. Materials and Methods

### 2.1. In Vitro Gastrointestinal Enzyme Digestion of Onion Peel Extract and Quercetin

Yellow onion peel (*Allium cepa* L.) was kindly provided from Boardman Foods Inc. (Boardman, OR, USA). It was washed twice with distilled water and dried overnight. The dried onion peels were used to make onion peel extract (OPE) according to a procedure previously described [22]. Onion peel extract (OPE) was stored at −20 °C for further use. OPE and Quercetin (Q) purchased from Sigma-Aldrich (Sigma-Aldrich, St. Louis, MO, USA) were digested using gastrointestinal digestive enzymes [23]. OPE or Q was acidified to pH 2 by 1 M HCl. Pepsin solution was added, and the mixture was incubated at 37 °C for 1 h. The mixture was adjusted to pH 6.9 by 1 M NaHCO_3_. Pancreatin-bile solution was added, and the mixture was incubated at 37 °C with shaking on a Solaris 2000 orbital shaker (Thermo scientific, Waltham, MA, USA) at 50 rpm for 2 h. The reaction was stopped by adding the amount of sodium chloride that referred to 3.5% of the total volume of the mixture. Solvents in the mixture were evaporated using a Rotavapor R-100 (BUCHI Corp, New Castle, DE, USA) to obtain digested onion peel extract (DOPE) or digested quercetin (DQ).

### 2.2. Quantification of Total Phenolic Compound and Quercetin

To compare the amount of quercetin before and after in vitro digestion of OPE, salt in DOPE was removed using Sep-Pak C18 Classic Cartridges (Waters Corp, Milford, MA, USA). Solid phase extraction samples (1 mg/mL) were analyzed using a Waters ARC HPLC system (Waters Corp, Milford, MA, USA) equipped with a Quaternary Solvent Management-R pump, Sample Manager FTN-R autosampler, and model 2998 photodiode array detector. Separation was carried out using a 4.6 mm × 250 mm Symmetry C18 column (Waters Corp, Milford, MA, USA) with a 3.9 mm × 20 mm Symmetry C18 guard column. The mobile phase was a linear gradient of 5% formic acid (A) and methanol (B) from 2% B to 60% B for 60 min. A detection wavelength of 360 nm was used for flavonols at a flow rate of 0.75 mL min^−1^. The injection volume was 50 µL. Data were expressed as mg quercetin equivalents (QE) per gram against a quercetin standard curve. Total phenolic content (TPC) of OPE and DOPE was measured using Folin–Ciocalteu’s phenol reagent as previously described [24].

### 2.3. Co-Culture of HT-29 Cells and Caco-2 Cells

HT-29 (ATCC HTB-38) and Caco-2 (ATCC HTB-37) cells were purchased from the American Type Culture Collection (ATCC, Manassas, VA, USA). The cells were maintained in Dulbecco’s Modified Eagle’s Medium (DMEM) supplemented with 10% fetal bovine serum, 1% non-essential amino acids, and 1% penicillin and streptomycin in a cell culture incubator with 5% CO_2_ at 37 °C. The cytotoxicity of OPE, DOPE, Q, DQ, and LPS was determined using 3-(4,5-dimethylthiazol-2-yl)-2,5-diphenyltetrazolium bromide (MTT) cell proliferation assay kit (Cayman, Ann Arbor, MI, USA) [18]. Caco-2 and HT-29 cells were mixed in a ratio of 9:1 and seeded into the apical chambers of 12-well Transwell plates at a density of 1 × 10^5^ cells/cm^2^. Cells were differentiated into a monolayer of cells for 21 days. Transepithelial electrical resistance (TEER) was measured every day. When TEER values Caco-2 and HT-29 co-cultures reached > 300 Ω × cm^2^ (~21 days after seeding), cells were incubated with OPE and DOPE (20, 30, and 60 µg/mL) and Q and DQ (5, 10, 20 µg/mL) for 12 h and then stimulated with 2 µg/mL LPS under the continuous treatment of these samples for the next 12 h.

### 2.4. Intestinal Permeability Assay

To measure the paracellular permeability across the co-culture monolayer as previously described [25], a mixture of 1 mg/mL fluorescein isothiocyanate (FITC, 4 kDa) and 0.1 mg/mL lucifer yellow (LY) (Sigma-Aldrich, St. Louis, MO, USA) were added to cells in the apical chamber of the Transwell. Cells were incubated with shaking at 150 rpm in the culture incubator at 37 °C for 12 h. To measure the amount of FITC and LY that were transferred from the apical side into the basal side, 100 mL of medium was taken from the basolateral side every hour, and then 100 mL of fresh medium was added to the basal side. The fluorescence was measured using a SpectraMax M3 fluorescence microplate reader (Molecular Devices, San Jose, CA, USA) with an excitation wavelength of 485 nm and 428 nm and an emission wavelength of 530 nm and 540 nm for FITC and LY, respectively. The apical-to-basal permeability coefficients (Pc) were calculated according to the equation: P(cm/s) = dQ/dT × (Vr/A × C_o_). Vr is the volume of the basal chamber (mL), A is the area of the membrane insert (cm^2^), and C_o_ is the apical donor concentration at the start of the experiment. T is the assay time (seconds).

### 2.5. Quantitative Polymerase Chain Reaction Analysis

Total RNA was isolated from Caco-2/HT-29 cells using TRIzol reagent and then was reverse-transcribed to synthesize cDNA using the GoStrip reverse transcription system (Promega, Madison, WI, USA) [18]. Gene expression was measured using the Fast Start Essential DNA SYBR Green Master Mix Kit in a Light Cycler 96 (Roche, Indianapolis, IN, USA). Ribosomal protein L32 (Rpl32) (Forward 5′-CCATCTCCTTCTCGGCATCAT-3′, Reverse 5′-AGCACTTCCAGCTCCTTGAC-3′) as a housekeeping gene was designed using Primer-BLAST (NCBI).

### 2.6. Western Blot Analysis

Total proteins were extracted from fully differentiated intestinal cells using RIPA lysis buffer. To determine NFκB activation, cytoplasm and nuclear were separated using a NE-PER Nuclear and Cytoplasmic Extraction Reagent Kit (Thermo Fisher Scientific, Waltham, MA, USA) according to the manufacturer’s instructions [18]. Western blot analysis was performed as previously described [18]. Primary antibodies against the following proteins were used: pAMPK, AMPK, ZO-1, CLDN1, SIRT1, and NFκB p65 (1:1000; Cell Signaling, Danvers, MA, USA) and β-actin (1:3000; Sigma-Aldrich). The following horseradish peroxidase-conjugated secondary antibodies were used: sheep anti-rabbit IgG (1:5000) for pAMPK, AMPK, ZO-1, CLDN1, SIRT1, and NFκB p65 (Novus Biology, Littleton, CO, USA), and goat anti-mouse IgG (1:5000) for β-ACTIN (Thermo Fisher Scientific). The target proteins were detected using enhanced chemiluminescence (Thermo Fisher Scientific) in Amersham Imager 600 equipped with ImageQuant TL8.2 (GE Healthcare, Chicago, IL, USA). The density of each band was analyzed using the National Institutes of Health ImageJ program (Version 1.54).

### 2.7. Statistical Analysis

Data were analyzed using a one-way analysis of variance with Tukey’s post hoc test (GraphPad Prism 6.0, GraphPad Software, Inc., San Diego, CA, USA) and presented as mean ± standard deviation (SD). A *p*-value less than 0.5 was considered statistically significant.

## 3. Results

### 3.1. TPC and Bioactive Compounds of OPE and DOPE

TPC values of OPE and DOPE were 580.03 and 599.61 GAE mg/g, respectively. At 360 nm, 14 peaks were detected and identified in the OPE and DOPE (Supplementary Figure S1 and Supplementary Table S1). Quercetin and quercetin-4′-glucoside were found as major compounds in OPE and DOPE. Quercetin concentration in OPE and DOPE was 86.1 ± 0.4 mg/g and 160.8 ± 1.2 mg/g, respectively, while quercetin-4′-glucoside was 37.6 ± 0.5 mg/g and 52.6 ± 0.2 mg/g.

### 3.2. OPE, DOPE, Q, and DQ Increased TEER and Intestinal Permeability in LPS-Induced Intestinal Cells

**Figure 1:**
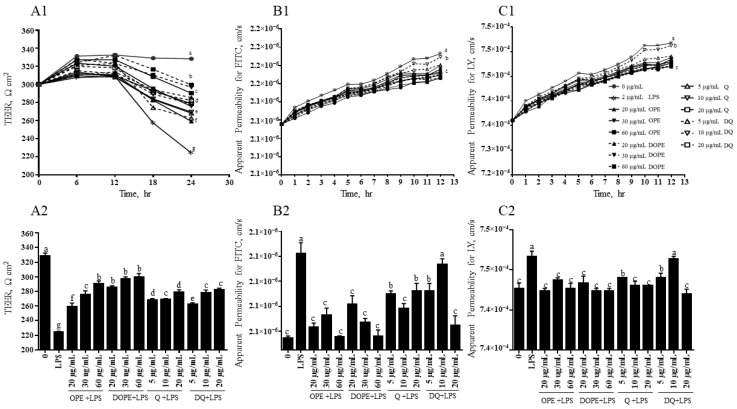
The effect of OPE, DOPE, Q, and DQ on transepithelial electrical resistance (TEER) and intestinal paracellular permeability in Caco-2/HT-29 cells. Caco-2/HT-29 cells were treated with OPE, DOPE, Q, and DQ for 12 h and then were induced by LPS together with OPE, DOPE, Q, or DQ for the next 12 h. (**A1**) TEER of cell monolayers from Caco-2/HT29 cells was determined every 6 h for 24 h of the sample treatment. (**A2**) TEER of cell monolayers from Caco-2/HT29 cells at the endpoint (24 h) of the sample treatment. (**B1**) Permeability was determined by measuring FITC-dextran that passed through the Caco-2/HT-29 every hour for 12 h after LPS and sample treatment. (**B2**) Permeability of FITC-dextran at the 12 h endpoint from the LPS and sample treatment. (**C1**) Permeability was determined by measuring the LY that passed through the Caco-2/HT-29 every hour for 12 h after LPS and sample treatment. (**C2**) Permeability of LY at the 12 h endpoint from the LPS and sample treatment. The experiment was performed in quadruplicate. Alphabetic letters indicate a significant difference (*p* < 0.05) between treatments.

LPS decreased TEER values and increased the paracellular permeability of the membrane in Caco-2/HT-29 cells (Figure 1). OPE and DOPE at 20, 30, and 60 µg/mL increased TEER by 15%, 23%, and 29% and 27%, 32%, and 33%, respectively (Figure 1A1). TEER was also increased by 19%, 20%, and 24% and 17%, 24%, and 25% by Q and DQ at 5, 10, and 20 µg/mL, respectively. Q and DQ did not show a difference at each concentration. In Figure 1B1, LPS increased the permeability of FITC by 53%, but 20, 30, and 60 µg/mL of OPE and DOPE reduced the LPS-increased permeability by 24%, 29%, and 32% and 29%, 25%, and 35%, respectively. There was no significant difference between OPE and DOPE at each concentration. Q at 5 µg/mL showed a higher decrease in the LPS-increased permeability than 5 µg/mL DQ (Q, 20%; DQ, 6%). Q and DQ at 10 and 20 µg/mL reduced 26% and 6%, 15%, and 29%, respectively, without significant difference between Q and DQ at each concentration. ×LPS also increased the permeability of LY by 38% (Figure 1C1). The LY permeability was decreased by 29%, 25%, and 28% and 24%, 30%, and 29% by OPE and DOPE at 10, 30, and 60 µg/mL. There was no significant difference between OPE and DOPE at each concentration. Although 5 µg/mL Q showed a 21% reduction, DQ did not affect the LPS-induced LY permeability. Q and DQ at 10 and 20 µg/mL significantly reduced LPS-induced LY permeability by 23% and 24% and 16% and 29%, respectively.

### 3.3. OPE, DOPE, Q, and DQ Increased Genes Related to Tight Junctions in Intestinal Cells

**Figure 2:**
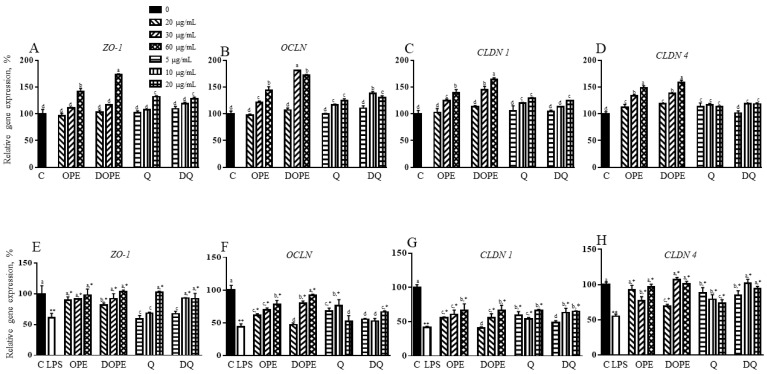
The effect of OPE, DOPE, Q, and DQ on genes related to TJ in Caco-2/HT-29 cells. (**A**–**D**) the expression of TJ genes in Caco-2/HT-29 cells treated with OPE, DOPE, Q, and DQ. (**E**–**H**) The expression of TJ genes in LPS-induced Caco-2/HT-29 cells with OPE, DOPE, Q, and DQ. The experiment was performed in triplicate. Alphabetic letters indicate a significant difference (*p* < 0.05) between treatments. ** control vs. LPS, * LPS vs. treatment.

OPE, DOPE, Q, and DQ increased genes encoding proteins related to tight junctions, including *ZO1*, *OCLN*, *CLDN 1*, *AND CLDN 4* (Figure 2A–D). OPE or DOPE at 60 µg/mL and Q or DQ at 20 µg/mL increased *ZO1* gene expression by 42% and 73% and 32% and 27%, respectively (Figure 2A). In Figure 2B, 30 µg/mL and 60 µg/mL of OPE and DOPE increased the *Ocln* gene by 21% and 44% and 81% and 72%, respectively. *OCLN* gene was also increased by 10 µg/mL and 20 µg/mL of Q and DQ. *CLDN 1* gene was upregulated by 30 µg/mL and 60 µg/mL of OPE and DOPE, with higher induction by DOPE than OPE (Figure 2C). Q at 10 µg/mL and 20 µg/mL and DQ at 20 µg/mL increased *CLDN 1* gene expression. As shown in Figure 2D, OPE and DOPE at 30 µg/mL and 60 µg/mL increased the *CLDN 4* gene by 33% and 49% and 38% and 59%. DQ at 10 µg/mL and 20 µg/mL induced this gene, but Q did not affect the gene.

LPS suppressed tight junction genes (Figure 2E–H). LPS suppressed the *ZO-1* gene by 39%, which was reversed to the control level by OPE and DOPE (Figure 2E). OPE showed better protection than DOPE at 20 µg/mL. DQ at 10 and 20 µg/mL reversed the LPS-suppressed *ZO-1* gene to the control, but Q showed the same effect at only 20 µg/mL. LPS-suppressed *OCLN* exhibited a 40%, 60%, and 77% increase by 20, 30, and 60 µg/mL OPE (Figure 2F). DOPE at 30 µg/mL increased *OCLN* gene expression by 80%. DOPE at 60 µg/mL protected the LPS suppression. The gene was also induced by 56% and 75% by 5 and 10 µg/mL Q, but it was not changed by 20 µg/mL Q. DQ at 20 µg/mL increased the *OCLN* gene by 22%. LPS suppressed the *CLDN 1* gene by 59% (Figure 2G). OPE and DOPE at 60 µg/mL similarly increased *CLDN 1* by 60%. Q at 5 and 20 µg/mL and DQ at 5 and 20 µg/mL also showed a similar increase. LPS-suppressed *CLDN 4* gene was protected by OPE at 20 and 60 µg/mL and OPE at 30 and 60 µg/mL (Figure 2H). The gene was significantly increased by 61%, 44%, and 34% and 54%, 86%, and 71% by 5, 10, and 20 µg/mL of Q and DQ, respectively.

### 3.4. OPE, DOPE, Q, and DQ Decreased the Expression of Genes Related to Inflammation in LPS-Induced Intestinal Cells

**Figure 3:**
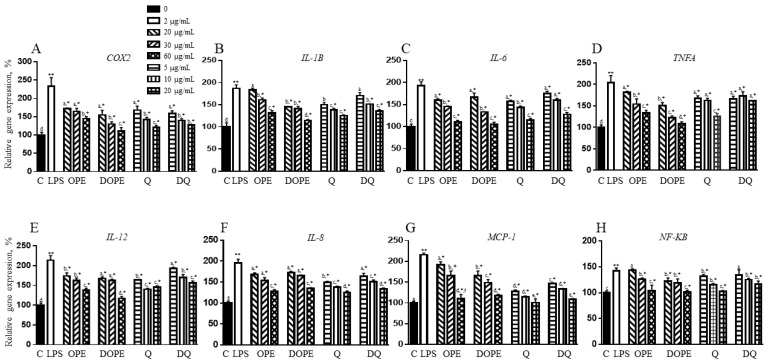
The effect of OPE, DOPE, Q, and DQ on genes related to proinflammatory cytokines in LPS-induced Caco-2/HT-29 cells. The expression of (**A**–**F**) proinflammatory cytokines genes, (**G**) macrophage marker gene, and (**H**) inflammatory transcription factor marker gene was determined using qPCR. The experiment was performed in triplicate. Alphabetic letters indicate a significant difference (*p* < 0.05) between treatments. ** control vs. LPS, * LPS vs. treatment.

LPS increased the Cox2 gene by 133%, but 20, 30, and 60 µg/mL of OPE and DOPE reduced the expression by 26%, 29%, and 38% and 34%, 44%, and 53%, respectively, with a better protective effect of DOPE at 30 and 60 µg/mL (Figure 3A). Q and DQ also showed a similar decrease, but there was no significant difference between Q and DQ. LPS-increased *IL1B* gene was decreased by OPE at 30 and 60 µg/mL and DOPE at 20, 30, and 60 µg/mL (Figure 3B). Q at 5, 10, and 20 µg/mL reduced the gene by 20%, 26%, and 30%, respectively. DQ at 5 µg/mL did not change the LPS-induced expression of the *IL1B* gene, whereas 10 and 20 µg/mL of DQ decreased *IL1B* expression by 19% and 27%, respectively. When the cells were treated with LPS, *Il6* gene expression was increased by 93% (Figure 3C). The gene was reduced by OPE and DOPE in a dose-dependent manner. OPE and DOPE at 20 µg/mL completely protected the LPS-induced change. The gene was also reduced by 19%, 26%, and 38% and 10%, 17%, and 34% by Q and DQ at concentrations of 5, 10, and 20 µg/mL, respectively. Q and DQ at 20 µg/mL reversed the LPS-induced *IL6* gene expression to the control level. The LPS-induced-*TNFA* gene was also reduced by 11%, 25%, and 34% and 25%, 40%, and 44%, by 20, 30, and 60 µg/mL OPE and DOPE, respectively (Figure 3D), showing a higher protective effect of DOPE than OPE. Q at 5 and 10 µg/mL and DQ at 5, 10, and 20 µg/mL showed a similar decrease in the LPS-induced *TNFA* gene. However, Q showed a higher reduction than DQ at 20 µg/mL. In Figure 3E, LPS induced *IL12* gene expression by 113%, but both OPE and DOPE reduced the expression, with the better effect of DOPE compared to OPE at each concentration. Q at 5 and 10 µg/mL showed a 23% and 34% decreased expression, but there was no additional reduction at 20 µg/mL. DQ at 5, 10, and 20 µg/mL decreased the LPS-induced *IL12* gene by 9%, 20%, and 27%, respectively. There was a significant difference between 5 and 10 µg/mL Q and DQ, but not at 20 µg/mL. As shown in Figure 3F, the cells treated with OPE and DOPE at 10 and 30 µg/mL showed a similar protective effect against LPS in the *IL8* gene. OPE and DOPE at 60 µg/mL reduced the LPS-induced *IL8* gene expression by approximately 60%. Q and DQ at 10 and 20 µg/mL exhibited a similar protective effect at each concentration. In Figure 3G, the LPS-induced *MCP-1* gene was reduced by OPE and DOPE in a dose-dependent manner. Q and DQ showed higher protective effects on the *MCP-1* gene against LPS than OPE and DOPE. Consistent with LPS-stimulated inflammatory markers, the *NF-KB* gene, a key inflammatory regulator, was induced by 42% when compared to the control (Figure 3H). OPE at 20 µg/mL did not change the LPS-induced *NF-KB* gene. However, the gene was reduced by OPE at 30 µg/mL and DOPE at 20 and 30 µg/mL. Both OPE and DOPE at 60 µg/mL reversed the LPS-induced *NF-KB* gene to the control level. Q and DQ reduced the LPS-increased *NF-KB* gene, but Q showed a better effect than DQ. OPE, DOPE, Q, and DQ did not change genes related to inflammation in cells that were not stimulated with LPS (Supplementary Figure S2).

### 3.5. OPE, DOPE, Q, and DQ Regulate Inflammation Through the AMPK-NFκB Signaling

**Figure 4:**
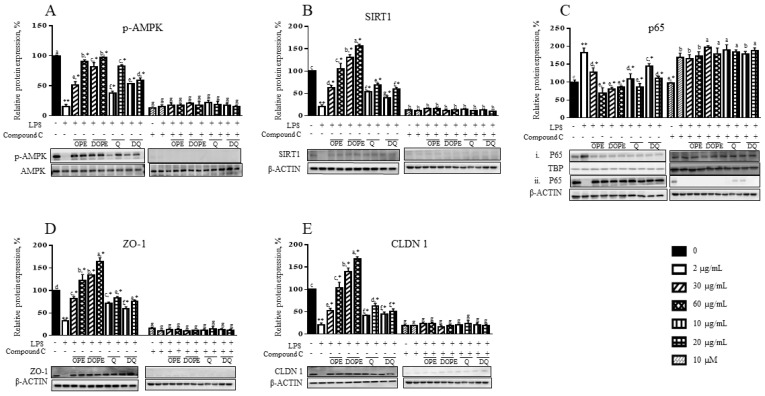
The effect of OPE, DOPE, Q, and DQ on proteins related to TJ and inflammatory regulation in LPS-induced Caco-2/HT-29 cells. (**A**) *p*-AMPK, (**B**) SIRT1, (**C**) NF-κB p65, (i) the nucleus and (ii) cytoplasm, (**D**) ZO-1, and (**E**) CLDN1 proteins were determined in the LPS-induced Caco-2/HT-29 cells with or without compound C (dorsomorphin). The lower panel of each graph shows a representative western blot image. (**A**) The *p*-AMPK was normalized by AMPK. (**B**) SIRT1, ZO-1, and CLDN were normalized by β-ACTIN. Nuclear and cytosolic p65 proteins were normalized by TBP and β-ACTIN, respectively. Alphabetic letters indicate a significant difference (*p* < 0.05) between treatments. ** control vs. LPS, * LPS vs. treatment.

To determine the mechanism by which quercetin exerts anti-inflammatory effects on LPS-treated cells, signaling proteins that regulate inflammation, adenosine monophosphate-activated protein kinase (AMPK), NF-κB, and their downstream proteins were examined (Figure 4). LPS dephosphorylated AMPK by 85% (Figure 4A). However, OPE and DOPE at 30 µg/mL showed 51% and 81% phosphorylation of AMPK compared to a control level. The LPS-suppressed AMPK phosphorylation was recovered by 60 µg/mL OPE and DOPE up to 91% and 97% of the control level, respectively. At 30 µg/mL, DQ had a higher protective effect than Q, but it was vice versa at 60 µg/mL. Under the treatment of compound C (dorsomorphin), an AMPK inhibitor, OPE, DOPE, Q, and DQ could not induce phosphorylation of AMPK.

SIRT1 protein level was suppressed in LPS-induced cells by 79% (Figure 4B). OPE at 30 µg/mL increased the SIRT1 protein level up to 63% of the control. The LPS-reduced SIRT1 was recovered by 60 µg/mL OPE to the control level. Interestingly, 30 and 60 µg/mL DOPE completely recovered the LPS-reduced SIRT1 protein level and showed an additional 30 and 57% increase in the SIRT1 protein compared to the control. Q and DQ also improved the LPS-reduced SIRT1 protein level. Q showed a better effect than DQ at each concentration. These protective effects against LPS were not observed in the cells treated with a compound C. LPS caused translocation of cytosolic NF-κB p65 subunit into the cell nuclear (Figure 4C). The LPS-induced nuclear translocation of p65 was decreased by 30% in the cells treated with 30 µg/mL OPE. OPE at 60 µg/mL and DOPE at 30 and 60 µg/mL reversed it to the control level. Q at 10 and 20 µg/mL reduced p65 nucleus translocation by 40% and 53%, whereas DQ decreased 20% and 39%, respectively. Under the compound C treatment, the LPS-induced nuclear translocation of p65 was not changed by OPE, DOPE, Q, and DQ. Consistent with the data observed in Figure 2, ZO-1 protein levels were decreased by LPS, which was recovered by 30 µg/mL OPE to 82% of the control (Figure 4D). OPE at 60 µg/mL completely protected the LPS-decreased ZO-1 protein and showed an additional 21% increase compared to the control. DOPE also exhibited an additional 34% and 65% induction with full protection against LPS in the ZO-1 protein. Q and DQ at 10 and 20 µg/mL recovered the ZO-1 protein to 72% and 83% and 59% and 75% of the control, respectively, but Q and DQ did not show a difference at each concentration. CLDN 1 protein was suppressed in LPS-induced cells by 80% (Figure 4E). Although 30 µg/mL OPE increased the LPS-decreased CLDN 1 protein, the protein was fully recovered by 60 µg/mL OPE. DOPE at 30 and 60 µg/mL completely reversed the LPS-decreased CLDN 1 protein and then showed an additional 40% and 69% increase in the protein (Figure 4E). Q and DQ at 30 µg/mL showed a similar increase in the LPS-decreased CLDN 1 protein, whereas Q had a higher increase than DQ at 60 µg/mL. These protective effects of OPE, DOPE, Q, and DQ on ZO1 and CLDN 1 were eliminated by compound C (Figure 4D,E).

## 4. Discussion

The intestinal system is a crucial interface with the external environment and is responsible for nutrient absorption and protection against harmful substances and microorganisms [5]. Maintaining intestinal homeostasis is vital for overall health, and it relies on the interplay of the intestinal epithelium, gut microbiome, and host immune system [5]. This intricate balance hinges on the integrity of gut epithelium barriers, upheld by junctional proteins such as TJ proteins, desmosomes, and adherent junctions [5]. These proteins form a physical barrier and connect adjacent epithelial cells [4]. Factors like gut microbiome composition, hormones, diet, and inflammatory mediators can influence these barriers [5]. When disrupted, these barriers can lead to uncontrolled passage of bacteria and their toxic byproducts, triggering a cascade of inflammatory responses in the bloodstream [4]. This is associated with the development of various diseases such as obesity, non-alcoholic fatty liver disease, cardiovascular diseases, inflammatory bowel disease, type 2 diabetes mellitus, and several autoimmune conditions [5]. Several anti-inflammatory drugs have been used to treat intestinal inflammation, including nonsteroidal anti-inflammatory drugs (NSAIDs) like ibuprofen and aspirin and corticosteroids like prednisone [26,27]. However, these medications have caused significant side effects, especially with long-term use, including renal injury, hepatotoxicity, respiratory tract inflammation and infection, gastric mucosal, and small bowel injuries [26]. Therefore, there is a growing interest in discovering alternative natural sources without side effects, particularly those involving edible plant-based bioactive compounds such as resveratrol from grapes, 6-gingerol from ginger, resveratrol from red wine, chlorogenic acid from coffee, and berberine from barberry, to reduce inflammation and improve gut barrier function [7,8,9,11]. Onion peel is a byproduct of onion but has shown high anti-inflammatory and antioxidant effects in vitro because it is high in quercetin [28,29,30]. Although some studies have shown the anti-inflammatory effects of onion peel on intestinal health in vitro, there is a lack of evidence to demonstrate whether the in vitro bioactivity of onion peel and quercetin has the same biological function in the in vivo system. Therefore, this study aimed to investigate the anti-inflammatory effects of in vitro gastrointestinal digested onion peel and quercetin, and their impact on the integrity of the intestinal barrier in comparison to the effects of intact onion peel and quercetin in Caco-2/HT-29 cells.

The LPS produced by harmful bacteria induces the phosphorylation of IκBα, leading to its degradation, which translocates NF-κB p65 and p50 from the cytoplasm to the nucleus [31]. In the nucleus, p65 upregulates the transcription of proinflammatory mediators and cytokines, such as COX-2, IL-6, and TNF-α [31]. TNF-α and IL-1β stimulate the production of other proinflammatory cytokines, IL-6 and IL-18, and neutrophil chemokines, IL-8 [32,33]. MCP-1 is a chemotactic factor inducing monocyte migration and macrophage differentiation. Overproduction of MCP-1 contributes to the production of TNF-α and the recruitment and activation of immune cells [34], accelerating inflammation. In the present study, OPE, DOPE, Q, and DQ reduced LPS-induced inflammation in Caco-2/HT-29 cells, evidenced by reduced *COX2*, *IL-1B*, *IL-6*, *TNFA*, *IL-12*, *IL-8*, and *MCP-1* expression. OPE and DOPE fully protected NF-κB p65 translocation at 60 µg/mL and Q and DQ at 20 µg/mL, suggesting quercetin reduced inflammation by regulating NF-κB. Acute necrotizing pancreatitis (ANP) is a severe condition that induces intestinal injury, characterized by disruption of the intestinal barrier and inflammation, promoting bacterial translocation and exacerbating systemic inflammatory responses [35]. Administration of quercetin (50 mg/kg body weight) via intraperitoneal injection to ANP rats for 6 and 12 h significantly reduced ileal epithelium intestinal inflammation and injury by downregulating proinflammatory cytokines IL-1β, TNF-α, and IL-17-α, primarily via decreasing the protein level of Toll-like receptor 4 (TLR4)**,** myeloid differentiation primary response 88 (MyD88), and inhibiting the activation of p38 mitogen-activated protein kinase (p38 MAPK) [35]. Quercetin also suppressed endoplasmic reticulum (ER) stress by inhibiting the unfolded protein response (UPR), evidenced by reduced levels of ER stress markers, including binding immunoglobulin protein, phosphorylated inositol-requiring enzyme 1 alpha, spliced X-box binding protein 1, phosphorylated eukaryotic initiation factor 2 alpha, and activating transcription factor 6 [35]. Although 40 and 80 µg/mL of onion peel extract prepared using 50% ethanol (AP50E) reduced IL-1β and IL-6 genes in LPS-induced RAW264.7 cells, AP50E did not change TNFα gene expression, NFκB/IκB α activation, and MAPK signaling [36]. Compared to our findings, this discrepancy may be due to the higher ethanol percentage we used to extract bioactive compounds, potentially yielding a higher amount of bioactive compounds and different susceptibility to different types of cells (Caco-2/HT-29 cells vs. RAW264.7 cells). Therefore, onion peel extract’s anti-inflammatory effects may vary based on extraction methods, cell types, and experimental conditions.

MCP-1 plays a crucial role in recruiting monocytes, macrophages, and other immune cells to sites of inflammation that secrete various enzymes, such as proteases and hydrolytic enzymes, which can break down cell membranes and extracellular matrix components [37]. While this enzymatic activity is important for immune function, such as eliminating pathogens and clearing damaged tissue, excessive release can lead to unintended damage to healthy cells and tissues [38]. MCP-1 mediates this process by binding to CCR2 receptors on monocytes and macrophages [39]. These activated immune cells can produce reactive oxygen species (ROS) through mechanisms such as the NADPH oxidase complex. While ROS play a role in normal immune function, their excessive production during inflammation can contribute to oxidative stress and tissue damage [39]. Although we did not examine the effect of our samples on inflammation-induced ROS, OPE (1–100 μg/mL) reduced ROS, H_2_O_2_, and hydroxynonenal-induced DNA damage in human-induced oxidative stress leukocytes [40]. Quercetin (25 μM) protected H_2_O_2_-exposed Caco-2 cells by reducing ROS and apoptosis through increased glutamate-cysteine ligase catalytic subunit, the rate-limiting enzyme in glutathione synthesis, leading to elevated intracellular glutathione levels [41]. Quercetin also exhibited protective effects against H_2_O_2_-induced cellular damage by potentially inhibiting the expression of aquaporin-3 (AQP3), a channel that facilitates H_2_O_2_ entry into cells, thereby reducing intracellular H_2_O_2_ levels. Simultaneously, quercetin upregulated NADPH oxidase 1 (NOX1), which increased extracellular H_2_O_2_ generation [41]. In vivo, onion peel water extract protects against indomethacin-induced oxidative stress by increasing Nrf2, a transcription factor that regulates cellular redox homeostasis, and the expression of antioxidant enzymes heme oxygenase-1 and NAD(P)H-quinone oxidoreductase 1 (NQO1) in the duodenal mucosa of indomethacin-treated rats [13]. This suggests that onion peel and quercetin may improve intestinal inflammation by preventing oxidative stress.

TJ proteins are essential multi-protein complexes that act as selective/semipermeable barrier that facilitates the transport of ions and solutes, help maintain cellular polarity, and prevent the translocation of luminal antigens, microorganisms, and their toxins into the body [4]. Claudins and occludin regulate ionic selectivity of the paracellular pathway and selective paracellular permeability, respectively [4]. ZO-1 connects transmembrane proteins to the actin cytoskeleton in the cell, recruiting other tight junction proteins to maintain intestinal function [4]. LPS interacts with immune cells, releasing proinflammatory cytokines that result in the phosphorylation of ZO-1, a protein associated with the actin cytoskeleton and helps anchor tight junctions’ protein, leading to cytoskeletal rearrangement, degrading of the tight junction structure and increased permeability [42,43]. This increases the development of abnormal conditions such as insulin resistance, inflammatory bowel diseases, obesity, and some cancers [5]. Thus, it is important to reduce intestinal inflammation and maintain the integrity of the intestinal barrier to promote gut health. In the present study, OPE and Q with or without digestion also increased TJ genes and proteins in the Caco-2/HT-29 cells, even though OPE and DOPE showed a more substantial effect than Q and DQ. This indicates that crude extract and a bioactive compound of onion peel can potentially improve intestinal tight junction by increasing TJ protein levels and the assembly of TJ proteins, but crude extract has a better effect on enhancing TJ protein than a single compound. Moreover, in the LPS-induced Caco-2/HT-29 cells, DOPE completely protected LPS-reduced ZO-1 and CLDN 1 and further increased their protein levels beyond the control level, but this change disappeared by dorsomorphin, indicating AMPK is an important mediator to maintain TJ proteins. This suggests that the digestive process may be critical in converting bioactive compounds in crude extract to more effective compounds. Inhibition of PKC increases AMPK activity by increasing its phosphorylation [44]. Consistent with our findings, Caco-2 cells treated with 30 and 100 µmol/L quercetin exhibited increased CLDN-1, CLDN-4, ZO-2, and OCLN proteins in the actin cytoskeleton compared to the cytosol, which increased TEER and decreased LY permeability by increasing dephosphorylation on Ser 643 of protein kinase C (PKC)δ that decreased PKCδ activity [45,46]. Amashed et al. showed that 100~200 µmol/L quercetin increased TEER by inducing CLDN 4 protein levels, but other TJ proteins were not increased in Caco-2 cells [46]. Myosin light chain kinase (MLCK) regulates TJ barrier structure and function by opening or lowering TJ protein assembly [47]. Preventing TJ barrier disruption requires inhibiting the MLCK/*p*-MLC pathway to maintain TJ integrity [47]. Quercetin and myricetin (2.5–20 μmol/L) with or without heat treatment, improved barrier function by increasing TEER and tight junction proteins (CLDN-1, CLDN-4, ZO-1) while reducing paracellular permeability and bacterial translocation by suppressing RhoA/ROCK signaling pathway, leading to suppression of phosphorylation of MLC and further contractility inhibition of the actinomyosin [48]. Therefore, onion peel extract and quercetin may regulate TJ proteins by a PKC-AMPK signaling pathway or a RhoA/ROCK-MLC pathway.

In the present study, LPS-decreased TJ genes were protected by OPE, DOPE, Q, and DQ. OPE, DOPE, Q, and DQ showed stronger protective effects on *ZO1* and *CLDN* 4 genes than *OCLN* and *CLDN 1*. Consistent with improved TJ genes and proteins, OPE, DOPE, Q, and DQ increased TEER and decreased permeability in LPS-stimulated Caco-2/HT-29 cells, suggesting both crude extract of onion peel and its single compound prevented inflammation-caused destructive process in the intestinal mucosa barrier. Consistent with our findings, the supplementation of 50 mg of quercetin per kg of body weight for 20 weeks improved microbiome diversity, which reduced LPS levels in the cecum of obese mice [49]. This alleviated LPS-decreased *MUCIN-2*, *OCLN*, *CLDN 1*, *CLDN 2*, and *ZO-1* expression in the intestine of obese mice, reduced serum LPS levels, and improved overall obese conditions [49]. Onion peel water extract and quercetin’s major metabolite of the water extract, 2-(3,4-dihydroxybenzoyl)-2,4,6-trihydroxy-3(2H)-benzofuranone (BZF) that naturally occurs in onion peels protected against indomethacin-induced decrease in TEER and increased permeability of FITC in Caco-2 cells by preventing NF-κB activation, which further decreased myeloperoxidase activity that catalyzes the production of potent ROS [30]. Quercetin (1 and 10 µM) treatment prevented IL-1β-induced permeability of FD-4 and TJ disruption in Caco-2 cells by inhibiting NF-κB p65 activation, ERK1/2 expression, and MLCK/*p*-MLC signaling pathway [50]. Quercetin (50 mg/kg body weight) protected intestinal barrier disruption by increasing ZO1, OCLN, and CLDN1 in the intestines of ANP-induced rats [35].

AMPK, an important regulator for the assembly and disassembly of TJ proteins in cell–cell junctions, enhanced epithelial differentiation and tight junction formation, which increased TEER and reduced paracellular FITC-dextran permeability in Caco-2 cells [51]. In the present study, LPS suppressed AMPK by dephosphorylation and reduced their downstream TJ proteins, ZO1, and CLDN1. The LPS-reduced AMPK activity was protected by OPE, DOPE, Q, and DQ in LPS-induced Caco-2/HT-29 cells, but interestingly, OPE and DOPE, but strongly by DOPE increased ZO1 and CLDN1 proteins to higher levels than their levels shown in the cells. These protective effects of OPE, DOPE, Q, and DQ disappeared when AMPK was inhibited. This suggests that OPE, DOPE, Q, and DQ regulated TJ proteins by regulating AMPK. However, additional induction on TJ proteins indicates that there may be another way to regulate TJ proteins in inflammation. SIRT1, a metabolic sensor, interacts with the p65 subunit of NF-κB and inhibits transcription by deacetylating p65 at lysine 310 [52]. In the present study, OPE, DOPE, Q, and DQ induced SIRT1 that inhibited p65 translocation of NF-κB and sequentially reduced inflammation, resulting in preventing LPS-caused tight junction damage in the LPS-induced Caco-2/HT-29 cells and this effect disappeared when AMPK was inhibited. Consistent with our findings, the treatment of pasteurized *Akkermansia muciniphila* decreased proinflammatory genes *TNF-α*, *IL-1α*, *IL-1β*, *IL-6*, and *IL-8* and increased TJ genes and proteins, resulting in reducing the permeability of FITC-dextran in LPS-induced Caco-2 cells by activating AMPK and inhibiting NF-κb through TLR 2 [53]. Therefore, OPE, DOPE, Q, and DQ protected intestinal tight junction against LPS-induced inflammation by directly activating AMPK and indirectly inhibiting NF-κB inflammatory signaling through AMPK-induced SIRT1.

Phenolic compounds are commonly found as glycosides or complexes linked to sugars, organic acids, amines, lipids, carbohydrates, and other phenols. Enzymatic treatments, such as the hydrolysis of starch and protein, can promote the release of these polyphenols [21]. The bioaccessibility of bioactive compounds, which refers to their liberation from the food matrix during digestion, is crucial in determining their biological properties. In the present study, OPE and DOPE showed higher anti-inflammatory effects on most inflammatory and TJ genes and proteins and intestinal mucosa barrier function than Q and DQ, suggesting crude extract may have better effects than single compounds by synergistic effects of compounds. In the last step of the enzymatic reaction to make DOPE, salt was added to stop the enzymatic reaction, which increased DOPE’s weight. So, the DOPE with salt had 50.7 mg quercetin/g. OPE and DOPE (20, 30, and 60 µg) that we treated in the cells included 1.72, 2.58, and 5.17 µg and 1.01, 1.52, and 3.04 µg quercetin due to salt, respectively, suggesting DOPE, even containing less quercetin/g compared to OPE had higher protective effects than OPE in the present study. When salt was removed from DOPE, the amount of quercetin in DOPE was increased to double the amount compared to OPE, indicating that gastrointestinal digestion may release aglycon by removing the glycosyl group. This suggests that DOPE may have much higher biological effects than OPE. In the present study, gastrointestinal digestion increased the levels of quercetin and quercetin-4-glucoside while decreasing their dimeric forms. This conversion from dimers to monomers may explain the increased bioactivity observed in DOPE. Moreover, quercetin concentrations in OPE and DOPE that we treated in the cells were less than concentrations of Q and DQ but had better effects. This indicates that gastrointestinal digestion may increase bioaccessibility and enhance synergistic effects from increased single compounds of OPE. Although intestinal quercetin concentrations or bioaccessibility of quercetin to the intestine is unclear, quercetin of OPE had better bioavailability (0.64%) than quercetin dihydrate (0.15%) [54]. Humans’ daily quercetin consumption ranges from 500 to 1000 mg. Therefore, a person consuming 1000 mg of OPE or quercetin may have 1.12~1.42 µg/mL of OPE or 0.26~0.33 µg/mL of quercetin in the blood when the adult human has 4500~5700 mL blood. Although intestinal cells have higher concentrations of bioactive compounds than blood due to bioavailability, our OPE concentrations used in the present study, similar to those in the blood, still showed anti-inflammatory and protective effects on the tight junction of the intestine.

There are some limitations in this study. First, because our in vitro digestion model demonstrated the protective effects of onion peel extract against LPS-induced intestinal inflammation, it did not account for gut microbiome interactions. The gut microbiota-derived glycosidase and esterase transform polyphenols through ring fission, while enzymes like chalcone isomerase and phloretin hydrolase further modify these compounds [55,56,57,58]. These microbial transformations can substantially alter the bioavailability and bioactivity of polyphenols. Second, this study did not consider the influence of gut hormones, such as glucagon-like peptide 1 (GLP-1), GLP-2, polypeptide YY, ghrelin, cholecystokinin, and serotonin (5-HT). These hormones play a role in controlling appetite, aiding in intestinal repair, regulating gut movement, and managing inflammation [59,60]. GLP-2 reduces intestinal inflammation and improves barrier function [61], while serotonin can impact immune responses in the gut [62]. Therefore, our findings may have limited applicability to real-life conditions where the interaction between polyphenols, gut hormones, and intestinal health is more complex. Future in vivo studies are warranted to comprehensively evaluate how these factors influence the anti-inflammatory properties of onion peel extract and quercetin in the intestine.

## 5. Conclusions

In conclusion, OPE and Q protect intestinal cell integrity by increasing TJ proteins against LPS-induced inflammation by directly increasing AMPK activity and by indirectly attenuating NF-κB activity through AMPK-SIRT1 signaling. Although gastrointestinal digestion did not reduce OPE or quercetin’s anti-inflammatory and TJ protective effects, crude extract has a better effect than a single compound, and digestion improves crude extract’s biological effects. Taken together, onion peel is a natural, sustainable, and cost-effective food byproduct that promotes intestinal health by improving intestinal integrity and reducing inflammation, which makes it an attractive alternative to conventional intestinal health supplements.

## Data Availability

The data presented in this study are available in the article and Supplementary Material. Further inquiries can be directed to the corresponding author on reasonable request.

## Supplementary Materials

The following supporting information can be downloaded at https://www.mdpi.com/article/10.3390/nu16213609/s1, Table S1: HPLC identification and quantification of phenolic compounds in onion peel extracts; Figure S1: HPLC chromatograms of bioactive compounds of OPE and DOPE. (A) Bioactive compounds of onion peel extract (OPE) identified at a wavelength of 360 nm,(B) Bioactive compounds of digested onion peel extract (DOPE) identified at a wavelength of 360 nm; Figure S2: The effect of OPE, DOPE, Q, and DQ on genes related to proinflammatory cytokines in Caco-2/HT-29 cells. The expression of (A–F) proinflammatory cytokines genes, (G) macrophage marker gene, and (H) inflammatory transcription factor marker gene was determined using qPCR. The experiment was performed in triplicate. Different alphabetic letters indicate a significant difference (*p* < 0.05) among groups.
