# Supplementary material for: Onion Peel Extract Prevents Intestinal Inflammation via AMK-Activated Protein Kinase Activation in Caco-2/HT-29 Cells
Source: Nutrients. 2024 Oct 24;16(21):3609. doi: 10.3390/nu16213609 (PMC11547908; doi:10.3390/nu16213609)
Supplement: Supplementary file 1 [file nutrients-16-03609-s001.zip › SupplementaryFiguresLegend.docx]

**Supplementary Figure Legends**

Supplementary Figure 1. HPLC chromatograms of bioactive compounds of OPE and DOPE

(A) Bioactive compounds of onion peel extract (OPE) identified at a wavelength of 360 nm,
(B) Bioactive compounds of digested onion peel extract (DOPE) identified at a wavelength of 360 nm

Supplementary Figure 2. The effect of OPE, DOPE, Q, and DQ on genes related to proinflammatory cytokines in Caco-2/HT-29 cells. The expression of (A-F) proinflammatory cytokines genes, (G) macrophage marker gene, and (H) inflammatory transcription factor marker gene was determined using qPCR. The experiment was performed in triplicate. Different alphabetic letters indicate a significant difference (*P* < 0.05) among groups.
