# Supplementary material for: Onion Peel Extract Prevents Intestinal Inflammation via AMK-Activated Protein Kinase Activation in Caco-2/HT-29 Cells
Source: Nutrients. 2024 Oct 24;16(21):3609. doi: 10.3390/nu16213609 (PMC11547908; doi:10.3390/nu16213609)
Supplement: Supplementary file 1 [file nutrients-16-03609-s001.zip › Supplementary Table 1.docx]

**Supplementary Table 1.** HPLC identification and quantification of phenolic compounds in onion peel extracts

| Peak | **Tentative Identification** | **OPE**  (mg/g extract) | **DOPE**  (mg/g extract) |
| --- | --- | --- | --- |
| 1 | Protocatechuic acid | 13.4 ± 0.0 | 39.4 ± 0.2 |
| 2 | 2-(3,4-dihydroxybenzoyl)-2,4,6-trihydroxy-3(2H)-benzofuranone | 3.1 ± 0.1 | 2.6 ± 0.0 |
| 3 | quercetin dihexoside | 1.0 ± 1.7 | 0.0 ± 0.0 |
| 4 | quercetin dihexoside | 0.5 ± 0.0 | 0.0 ± 0.0 |
| 5 | Quercetin-4'-glucoside | 37.6 ± 0.5 | 52.6 ± 0.0 |
| 6 | unknown peak | 0.5 ± 0.0 | 0.0 ± 0.0 |
| 7 | quercetin hexoside | 0.3 ± 0.0 | 2.3 ± 0.1 |
| 8 | Isorhamhex | 1.8 ± 0.1 | 136.6 ± 3.5 |
| 9 | protocatecoyl quercetin | 0.8 ± 0.0 | 0.5 ± 0.0 |
| 10 | quercetin | 86.0 ± 0.4 | 160.8 ± 1.2 |
| 11 | quercetin dimer 4 glucoside | 3.7 ± 0.3 | 2.2 ± 0.2 |
| 12 | quercetin dimer 4 glucoside | 6.3 ± 0.3 | 1.7 ± 0.1 |
| 13 | quercetin dimer | 11.5 ± 0.2 | 0.0 ± 0.0 |
| 14 | quercetin trimer | 14.7 ± 2.3 | 0.0 ± 0.0 |

OPE: onion peel extract; DOPE: digested onion peel extract
