# Supplementary figures and images for: Onion Peel Extract Prevents Intestinal Inflammation via AMK-Activated Protein Kinase Activation in Caco-2/HT-29 Cells
Source: Nutrients. 2024 Oct 24;16(21):3609. doi: 10.3390/nu16213609 (PMC11547908; doi:10.3390/nu16213609)

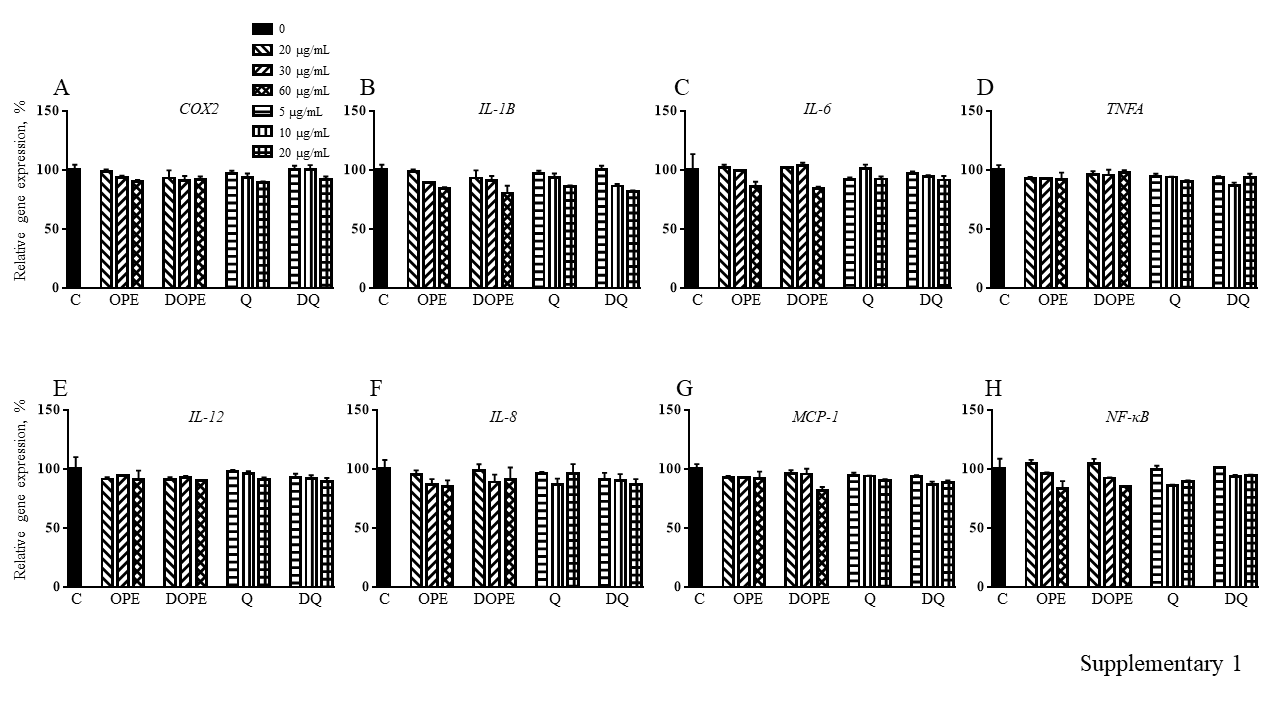

Supplement: Supplementary file 1 [file nutrients-16-03609-s001.zip › Supp Fig 2.TIF]

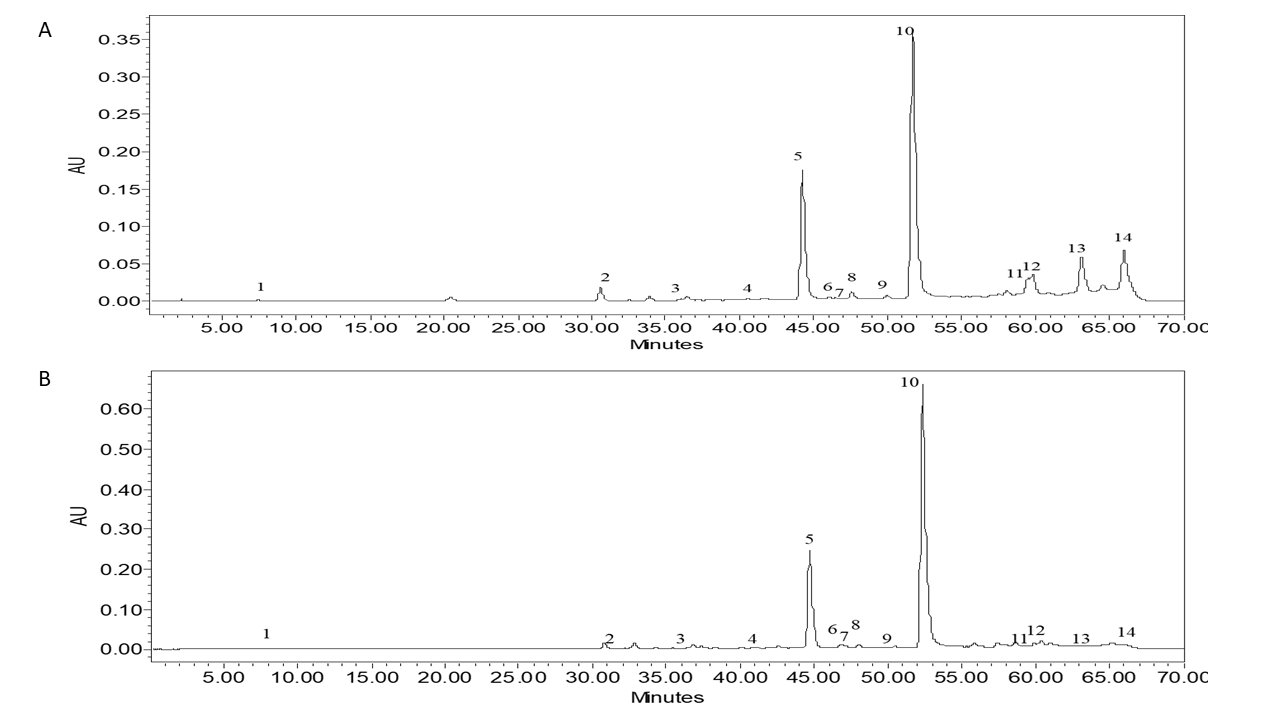

Supplement: Supplementary file 1 [file nutrients-16-03609-s001.zip › SuppleFigure 1.tif]
